# Intrinsic work values and proactive behavior among early-career employees: boundary conditions of perceived employment relations climate and internal locus of control

**DOI:** 10.3389/fpsyg.2026.1893310

**Published:** 2026-08-26

**Authors:** Jiangnan Hu, Jie Liu

**Affiliations:** 1 Yingtan Vocational and Technical College, Yingtan, China; 2 Department of Psychology, Wuhan University, Wuhan, China

**Keywords:** early-career employees, locus of control, perceived employment relations climate, proactive behavior, work values

## Abstract

**Introduction:**

Early-career employees are expected to learn their roles and to contribute proactively to organizational improvement. Although proactive behavior research has emphasized personality, leadership, job design, and socialization, less is known about how intrinsic work values are translated into proactive behavior among employees at the beginning of their careers. This study develops a value-based account of early-career proactivity and examines two boundary conditions: perceived employment relations climate and internal locus of control.

**Methods:**

Data were collected through the Wenjuanxing online questionnaire platform. Of 605 returned questionnaires, 541 valid responses from Chinese employees with no more than 24 months of work experience were retained. The survey measured intrinsic work values, proactive behavior, perceived employment relations climate, internal locus of control, and demographics. Reliability analyses, confirmatory factor analysis, correlation analyses, robust hierarchical regressions, moderation tests, simple-slope analyses, variance-inflation diagnostics, and Johnson-Neyman probing were conducted.

**Results:**

Intrinsic work values were positively associated with proactive behavior in the main-effect model. Perceived employment relations climate attenuated this association, such that intrinsic work values were more strongly related to proactive behavior when perceived employment relations climate was lower. Internal locus of control strengthened the association, such that intrinsic work values were more strongly related to proactive behavior among employees with higher internal locus of control.

**Discussion:**

The findings suggest that intrinsic work values are a motivational basis for early-career proactivity, but their behavioral expression depends on situational cues and perceived agency. The study contributes to value-behavior and proactive motivation research while highlighting the need for longitudinal and multi-source designs.

## 1 Introduction

Organizations increasingly depend on employees who can identify emerging problems, acquire information, improve work methods, and initiate constructive change before formal instructions are issued. This demand has made proactive behavior a central topic in organizational psychology. Proactive behavior differs from routine task performance because it is anticipatory, self-starting, and directed toward changing oneself, one’s role, or the work environment (Crant, 2000; Grant and Ashford, 2008). Recent Frontiers studies have examined proactive behavior under job insecurity and performance-appraisal arrangements, showing that initiative remains theoretically important in uncertain and evaluative work contexts (Yao et al., 2021; Yang et al., 2023). Other recent work has linked proactive work behavior to individual sensitivity, psychological empowerment, and career growth processes, further reinforcing the need to understand both personal and contextual antecedents (Schmitt, 2022; Ma et al., 2024; Satwika et al., 2025). Recent work on wise proactivity further emphasizes that initiative is most valuable when employees act with judgment rather than merely doing more (Wang et al., 2025). In uncertain and interdependent work systems, such behavior helps organizations adapt while also allowing employees to shape their own work trajectories (Parker and Collins, 2010; Parker et al., 2010).

The early-career period is a particularly important context for studying proactive behavior. Newcomers are not only learning assigned tasks; they are also interpreting organizational norms, building social relationships, seeking feedback, and deciding what kind of employee they will become. Classic socialization theory emphasized that organizational entry involves role learning and sensemaking (Van Maanen and Schein, 1979; Morrison, 1993). Later meta-analytic work showed that newcomer adjustment is shaped by both organizational tactics and newcomer agency (Bauer et al., 2007; Saks et al., 2007). More recent evidence further suggests that proactive newcomer behaviors, including information seeking and relationship building, are generally beneficial for socialization outcomes, and recent Frontiers research has likewise treated novice employees as a distinct group facing developmental self-regulatory demands (Zhao et al., 2023; Liu et al., 2026). These findings imply that early-career employees should not be viewed as passive recipients of socialization; they are active participants in constructing their work roles.

The theoretical issue, however, is not simply whether early-career employees can be proactive. A sharper puzzle is that proactive behavior is simultaneously adaptive and risky during early career entry. Although newcomers benefit from seeking feedback, improving work methods, and anticipating future demands, they also have limited local knowledge, uncertain legitimacy, and relatively little status from which to challenge routines (Morrison, 1993; Ashforth et al., 2007; Bauer et al., 2007; Pfrombeck et al., 2022). This tension makes early-career proactivity theoretically non-obvious: initiative can accelerate learning, but it can also expose employees to evaluation, rejection, or accusations of overstepping.

Prior research has explained this tension mainly through socialization tactics, proactive personality, job design, supervisor support, and self-efficacy. These perspectives are valuable, but they leave the value-based motivation for early-career initiative underspecified. If proactivity is treated only as a function of personality or organizational invitation, theory may overlook why some employees regard initiative as personally worth pursuing even when rewards are delayed, instructions are ambiguous, and acceptance is not guaranteed. The present study therefore asks how an intrinsic valuation of work as learning, challenge, growth, and meaningful accomplishment is associated with proactive behavior under different perceived contextual and agency conditions.

Intrinsic work values provide a theoretically meaningful answer to that question. Work values are relatively enduring beliefs about what people consider important, desirable, and worth pursuing in work. Early theories of vocational behavior treated work values as standards that guide occupational choice and work adjustment (Super, 1962; Dawis and Lofquist, 1984). Contemporary reviews similarly emphasize that values shape how employees evaluate work and decide which goals deserve effort (Arieli et al., 2020; Chang et al., 2024). Recent personnel research also suggests that value assessment remains relevant for understanding work-related choice and selection contexts (Anglim et al., 2022). Intrinsic work values refer specifically to the importance attached to growth, learning, challenge, autonomy, achievement, and meaningful work. They are related to intrinsic motivation, but they are not the same construct: intrinsic work values are evaluative beliefs about desirable work attributes, whereas intrinsic motivation describes the felt energization of behavior (Deci and Ryan, 2000; Ryan and Deci, 2000; Gagne and Deci, 2005).

This distinction is central to the present study. If proactive behavior were explained only by momentary interest or current job satisfaction, then value-based theorizing would add little. Intrinsic work values instead point to a deeper evaluative orientation: work is seen as a setting in which one ought to learn, develop competence, meet challenges, and realize personal potential. Such values may be especially consequential when formal job requirements do not specify what employees should improve or when immediate rewards for initiative are ambiguous. In those circumstances, values can provide a reason to act before the environment provides a clear instruction.

The focus on intrinsic work values is therefore not a convenient narrowing of a broad work-values scale; it is a theoretical choice. Different work-value dimensions imply different behavioral logics. Extrinsic values emphasize pay, security, and material return; status values emphasize prestige and recognition; interpersonal values emphasize harmonious relationships; leisure values emphasize flexibility and non-work time. These value domains may shape career preferences and work attitudes, but they do not map as directly onto the psychological requirements of proactive behavior. Proactivity requires employees to treat uncertainty, challenge, learning, and self-initiated change as worthwhile. Intrinsic work values are the dimension that most directly gives those demands positive meaning.

A values perspective is useful because proactive behavior is discretionary rather than merely compliant. Employees may choose to initiate proactive action without immediate rewards, clear instructions, or guaranteed acceptance. Under such conditions, values can help employees determine whether a behavior is worth the extra effort and interpersonal risk. Intrinsic work values are especially relevant because they define work as a domain for mastery, self-development, and meaningful accomplishment rather than only as a source of pay, status, or security.

However, value-based proactivity is unlikely to be automatic. Newcomers operate in organizational contexts that signal what behavior is safe, expected, and supported. Social information processing theory suggests that employees rely on contextual cues to interpret appropriate behavior at work (Salancik and Pfeffer, 1978). Climate theory similarly argues that shared or perceived organizational cues can strengthen or weaken the effect of individual differences by shaping behavioral norms (Schneider, 1975; Schneider et al., 2013). In the present study, perceived employment relations climate captures whether employees perceive labor-management relations as respectful, cooperative, and fair. This climate may either make proactivity broadly normative or leave employees to rely more heavily on their own internal values.

Value expression also depends on agency beliefs. Internal locus of control reflects the belief that one’s own effort and actions can influence outcomes (Rotter, 1966; Spector, 1988). Meta-analytic evidence links internal control beliefs to work attitudes, motivation, and active coping at work (Ng et al., 2006). More recent reviews suggest that locus of control remains relevant for understanding agency in organizational behavior, especially when employees face uncertain or demanding contexts (Galvin et al., 2018). For early-career employees, internal locus of control may determine whether intrinsic work values remain private preferences or become proactive action.

The two boundary conditions are therefore not merely statistical additions to a main-effect model. Perceived employment relations climate represents the external meaning system that tells employees whether initiative is legitimate in the organization, whereas internal locus of control represents the internal agency belief that tells employees whether their own initiative can matter. Considering both conditions allows the study to distinguish value endorsement from value enactment. An employee may strongly value growth-oriented work, but proactive behavior should be more likely when the employee either needs to rely on those values in a weaker climate or has sufficient agency beliefs to act on them.

This study therefore develops a person-context model of value-based proactivity. It examines whether intrinsic work values are associated with proactive behavior among Chinese early-career employees and whether this association depends on perceived employment relations climate and internal locus of control. The model is not intended to establish causal ordering from a single-wave survey; rather, it tests theoretically derived associations and boundary conditions that clarify when intrinsic work values are more or less strongly linked to proactive behavior.

## 2 Theoretical background and hypotheses

### 2.1 Intrinsic work values and proactive behavior

Work values are enduring evaluative standards concerning what people regard as desirable, important, and worth pursuing in their work. They are not momentary preferences for a particular job feature, nor are they identical to work motivation at a given point in time. Foundational vocational theories treated values as a central element of person-environment fit and work adjustment because values shape what employees seek from work and how they evaluate occupational experiences (Super, 1962; Dawis and Lofquist, 1984). Broader values research similarly argues that values provide trans-situational standards that help individuals select goals, interpret situations, and justify action (Schwartz, 1992; Bardi and Schwartz, 2003). In the present study, intrinsic work values refer to the importance employees attach to learning, growth, challenge, accomplishment, autonomy, and meaningful use of their abilities. In Chen’s Chinese work-values framework, this dimension is part of a broader system of Chinese employees’ work values, but it has a clear substantive focus on the developmental and self-realizing meaning of work (Chen, 2012).

The distinction between intrinsic work values and adjacent constructs is theoretically important. Intrinsic motivation describes the experienced enjoyment or inherent satisfaction of a current activity, whereas intrinsic work values describe whether employees believe that growth, learning, challenge, and meaningful accomplishment are important standards for evaluating work. Learning goal orientation emphasizes preferences for developing competence; proactive personality captures a broad dispositional tendency to change one’s environment; role-breadth self-efficacy concerns perceived capability to carry out broader tasks; career adaptability and future work self-concern career management and future-oriented identity. Intrinsic work values differ from these constructs because they specify what employees regard as worth pursuing in work, rather than whether they are generally change-oriented, confident, adaptable, or currently enjoying a task.

This construct specificity matters for the present model. A manuscript that used the total score of work values would blur together motives that may point in different behavioral directions. Extrinsic reward values may encourage performance when initiative is visibly rewarded, but they may not sustain self-starting action when rewards are delayed or uncertain. Interpersonal values may support cooperation, yet they may also discourage challenging established routines when harmony is threatened. Status values may encourage visible achievement, but they can also make employees cautious about failure. Intrinsic work values are distinctive because their content directly affirms the developmental experiences that proactive behavior often requires: learning from feedback, experimenting with improvements, accepting challenge, expanding competence, and constructing a meaningful professional identity.

The motivational force of intrinsic work values can also be understood through expectancy-value reasoning. Employees are more willing to invest effort when they attach importance to the goal being pursued and when the action is connected to valued aspects of the self (Eccles and Wigfield, 2002). Intrinsic work values make proactive behavior identity-relevant because taking initiative can demonstrate competence, deepen learning, and transform work into a setting for self-development. This is different from treating proactivity as a generic desirable behavior. For employees high in intrinsic work values, proactive behavior is not only useful for the organization; it is also congruent with what they believe good work should offer and what kind of professional they want to become.

Proactive behavior is a particularly relevant outcome for a values perspective because it is discretionary and future-oriented. Proactive employees do not merely respond to assigned duties; they anticipate problems, seek information, improve procedures, and attempt to change themselves or their environment before external demands require them to do so (Crant, 2000; Grant and Ashford, 2008). Closely related research on personal initiative similarly emphasizes self-starting, persistent, and change-oriented action at work (Fay and Frese, 2001; Frese and Fay, 2001). Meta-analytic work has further shown that the proactivity domain is conceptually broad, making it important to specify the motivational route being studied rather than treating all proactive constructs as interchangeable (Tornau and Frese, 2013). Such behavior is often beneficial, but it is also psychologically demanding. It may require employees to invest effort without immediate reward, challenge established routines, risk interpersonal discomfort, and continue acting even when outcomes are uncertain. Proactive motivation theory therefore emphasizes that employees need reasons to act, beliefs that they can act, and energy to sustain action (Parker et al., 2010; Strauss et al., 2012). Intrinsic work values speak directly to the first of these questions: they help explain why proactive action is experienced as personally meaningful rather than merely as extra work.

This argument also helps separate value-driven proactivity from simple compliance with organizational expectations. Employees can behave proactively because they are instructed to be innovative, because supervisors reward voice, or because the job provides autonomy. Those explanations are context-centered. The present argument is person-centered but not trait-reductionist: intrinsic work values provide an evaluative reason for action without assuming that all intrinsically oriented employees will behave proactively in every setting. This distinction is important because it allows the study to explain both the main association and the later boundary conditions. Values orient employees toward initiative, but contextual cues and agency beliefs determine whether that orientation is expressed.

This logic is especially compelling for early-career employees. Organizational entry is a period of role learning, identity construction, and social sense-making. Newcomers must learn formal tasks, decode informal norms, build relationships, and develop confidence in their ability to contribute (Van Maanen and Schein, 1979; Morrison, 1993). Socialization research has shown that newcomer adjustment depends not only on organizational tactics but also on employees’ own proactive efforts to seek feedback, acquire information, and build social connections (Bauer et al., 2007; Saks et al., 2007). When early-career employees strongly value growth and mastery, proactive behavior becomes a natural route for enacting these values. Seeking feedback can be understood as pursuing learning; proposing improvements can be understood as exercising competence; taking initiative can be understood as building a meaningful work identity.

Recent work on careers and employee development reinforces this argument. Contemporary careers require employees to adapt, learn continuously, and actively shape their work roles rather than relying exclusively on organizationally prescribed progression (Demerouti, 2023; Wang et al., 2024). Studies of proactive work behavior also suggest that meaningfulness, role-breadth beliefs, mentoring, and learning-oriented motivation are important antecedents of initiative (Parker et al., 2006; Griffin et al., 2007; Xu et al., 2021; Yang et al., 2024). Related evidence links proactive behavior to psychological empowerment, prosocial motivation, and proactive motivation in contemporary work settings (Iqbal et al., 2024; Xu F. et al., 2024; Xu Q. et al., 2024). For early-career employees, intrinsic work values should therefore operate as a motivational foundation that makes proactive behavior consistent with valued self-development. The present study accordingly expects intrinsic work values to be positively associated with proactive behavior.

*H1*: Intrinsic work values are positively associated with proactive behavior among early-career employees.

### 2.2 Perceived employment relations climate as a contextual moderator

Values do not operate in a vacuum. Whether an employee expresses a value through behavior depends partly on whether the work environment makes that behavior seem legitimate, safe, and useful. Perceived employment relations climate refers to employees’ perceptions of the quality of the relationship between labor and management, including communication, mutual respect, procedural fairness, cooperation, and the extent to which employees believe that management treats workers as valued organizational members. This construct is closely related to, but narrower than, broad organizational climate: it focuses on the perceived social contract between employees and the organization and on the relational tone that frames employee participation.

Perceived employment relations climate is especially relevant for early-career employees because they are still learning how much discretion they have and how management responds to employee initiative. A newcomer may value challenging and meaningful work, yet still hesitate if the employment relationship appears distant, adversarial, or purely transactional. Conversely, when employees perceive that managers communicate respectfully, keep promises, and consider employee perspectives, the climate communicates that the organization treats employees as legitimate contributors rather than replaceable labor. This relational signal is important because proactive behavior often requires employees to expose problems, suggest change, or act before formal authority has granted explicit permission.

Climate theory provides one foundation for expecting perceived employment relations climate to shape value expression. Organizational climate consists of psychologically meaningful cues that signal what the organization rewards, supports, and expects (Schneider, 1975; Schneider et al., 2013). Reviews of the climate literature emphasize that such cues become behaviorally consequential when they are coherent, salient, and attached to meaningful organizational practices (Bowen and Ostroff, 2004; Kuenzi and Schminke, 2009; Zohar and Hofmann, 2012). Social information processing theory similarly argues that employees interpret workplace cues to decide what attitudes and behaviors are appropriate in a given setting (Salancik and Pfeffer, 1978). For proactive behavior, such cues are crucial because initiative often involves stepping beyond minimum role requirements. Employees are more likely to speak up, seek changes, or invest extra effort when the environment suggests that such behavior will be heard rather than punished. Recent studies have similarly connected employment relationship atmosphere, ethical climate, and team climate with proactive or innovative work behavior (Fan L. et al., 2022; Fan X. et al., 2022; Gok et al., 2023; Ni and Zheng, 2024). Research on employee voice also shows that managerial endorsement and strategic silence shape whether constructive expression is valued (Burris et al., 2022; Parke et al., 2022). A favorable perceived employment relations climate can therefore reduce ambiguity about whether constructive initiative is acceptable.

The moderating direction expected in this study follows situational strength theory. Strong situations provide clear expectations, consistent incentives, and shared interpretations, thereby reducing the extent to which individual differences predict behavior (Mischel, 1977; Meyer et al., 2010). A favorable perceived employment relations climate may operate as such a strong situational cue. When labor-management relations are cooperative and respectful, proactive behavior may become broadly normative: even employees whose intrinsic work values are not especially strong may still feel invited to participate, offer suggestions, and seek improvement. In this situation, the behavioral difference between employees high and low in intrinsic values should narrow because the context itself supplies support for initiative.

By contrast, a weaker perceived employment relations climate may create a more ambiguous and less supportive setting. Early-career employees may not be sure whether management welcomes initiative, whether suggestions will be taken seriously, or whether taking action beyond formal duties could create interpersonal risk. In such situations, employees cannot rely as much on contextual encouragement. Their own values may become more diagnostic of behavior. Employees who strongly value learning, challenge, and meaningful accomplishment may still initiate change because proactive behavior expresses what they believe work should be about. Employees with weaker intrinsic values may be less likely to do so because the climate does not compensate for the absence of strong internal value commitments.

The hypothesized attenuation pattern is therefore theoretically different from a resource-enhancement pattern. A resource-enhancement argument would suggest that good employment relations should always strengthen the effect of positive personal attributes. The present argument instead emphasizes substitutability between contextual cues and personal values. When the climate already makes initiative legitimate, intrinsic values are not irrelevant, but they are less necessary for proactive behavior to occur. When the climate is weaker, intrinsic values become more important because they provide an internal standard for acting despite lower contextual encouragement. This logic is particularly suitable for explaining why supportive climates can raise overall proactivity while simultaneously reducing the predictive power of individual value differences.

This argument should not be misread as implying that a positive perceived employment relations climate is harmful. The expected negative interaction is an attenuation or compensatory pattern. A favorable climate may raise the general level of proactive behavior while reducing between-person differences in value-driven proactivity. A weaker climate may make intrinsic work values more visible because those values become a substitute source of direction when the situation provides fewer supportive cues. This interpretation is consistent with person-context interaction approaches, which emphasize that individual dispositions and contextual conditions often combine in non-additive ways (Tett and Burnett, 2003; Johns, 2006). The present study therefore expects the association between intrinsic work values and proactive behavior to be weaker when perceived employment relations climate is higher.

*H2*: Perceived employment relations climate moderates the association between intrinsic work values and proactive behavior, such that the association is weaker when perceived employment relations climate is higher.

### 2.3 Internal locus of control as a dispositional moderator

A second boundary condition concerns agency beliefs. We conceptualize internal locus of control as a moderator rather than as an antecedent or mediator because it represents a generalized contingency belief about whether one’s actions can influence outcomes, not a value content in itself. Intrinsic work values indicate what employees care about in work; internal locus of control indicates whether acting on those values seems personally consequential. The two constructs are related but not interchangeable: an employee may value growth and challenge yet still doubt that personal initiative will affect workplace outcomes.

Internal locus of control is useful here because it captures a general contingency belief rather than a narrow skill judgment. Constructs such as self-efficacy describe whether employees believe they can perform a specific task, whereas locus of control concerns whether outcomes are generally seen as connected to one’s own behavior (Lefcourt, 1982; Spector, 1988). Proactive behavior often unfolds before task demands are fully specified, especially for newcomers who are still learning what counts as effective contribution. In such situations, employees may not yet have strong task-specific efficacy beliefs, but a more internal control orientation can still support the belief that effort, persistence, and initiative are worthwhile.

Internal locus of control helps explain why the same value may or may not be associated with behavior. Values specify what is important; agency beliefs shape whether pursuing that value seems feasible and consequential. An early-career employee may value learning, challenge, and meaningful accomplishment, but if workplace outcomes are perceived as mainly controlled by luck, hierarchy, or opaque rules, proactive behavior may appear futile or risky. By contrast, when employees believe that their own actions can influence outcomes, intrinsic work values have a clearer route into information seeking, problem solving, and constructive change.

Trait activation theory provides a further explanation. Traits and trait-like dispositions are most likely to influence behavior when situations contain cues that make the disposition relevant (Tett and Burnett, 2003). Proactive behavior offers precisely such cues because it involves discretionary choice, uncertainty, and personal responsibility. These features activate control beliefs: employees must decide whether they can influence a problem, acquire useful information, or improve a work process. Internal locus of control should therefore amplify the effect of intrinsic work values by making value-consistent action feel controllable. In contrast, employees lower in internal locus of control may endorse intrinsic work values but be less likely to act on them when proactive behavior appears risky or unlikely to change outcomes.

This moderation is especially important among early-career employees because their organizational position is often ambiguous and relatively low in formal power. They may lack established status, deep social networks, and clear knowledge of organizational routines. Such conditions can make proactive behavior psychologically costly. Internal locus of control may help early-career employees persist despite these obstacles by supporting a sense of personal efficacy and responsibility. It may also encourage employees to interpret setbacks as information for adjustment rather than as evidence that initiative is futile. In this way, locus of control complements intrinsic work values: values provide direction, whereas internal control beliefs support enactment.

The proposed interaction also prevents an over-socialized account of newcomer behavior. Organizational climate and supervisor support matter, but early-career employees are not merely products of their environments. They differ in the extent to which they see themselves as agents capable of shaping outcomes. When internal locus of control is high, intrinsic work values should have more behavioral force because employees are more likely to treat initiative as a controllable route to growth. When internal locus of control is low, intrinsic values may still be endorsed, but the employee may wait for external direction, attribute outcomes to organizational constraints, or avoid investing effort in change attempts that seem unlikely to matter.

Recent discussions of agency at work continue to treat control beliefs as relevant for understanding how employees respond to uncertainty, change, and demanding work environments (Galvin et al., 2018; Ben-Meir et al., 2024; Wu et al., 2024). For the present model, the key claim is not that internal locus of control universally increases proactive behavior, but that it strengthens the behavioral expression of intrinsic work values. Employees high in intrinsic values and high in internal locus of control should be particularly likely to engage in proactive behavior because they both value growth-oriented work and believe that their own actions can shape work outcomes.

*H3*: Internal locus of control strengthens the positive association between intrinsic work values and proactive behavior.

### 2.4 Present study

At the same time, the model does not assume that intrinsic work values automatically become proactive behavior. Value enactment depends on whether the surrounding work environment makes initiative intelligible and whether the employee believes personal action can influence outcomes. Perceived employment relations climate captures the contextual meaning system surrounding employee-management relations. When this climate is favorable, respectful, and cooperative, proactive behavior may become a generally legitimate mode of participation, reducing the extent to which only highly intrinsically oriented employees engage in initiative. Internal locus of control captures the agency belief needed for value enactment. Employees who believe that outcomes are contingent on their own effort should be more likely to translate intrinsic values into action.

This design reflects a deliberate construct decision. Rather than treating work values as a broad omnibus predictor, the study focuses on intrinsic work values because this dimension has a direct bridge to theories of motivation, self-development, proactive work behavior, and career agency. The resulting model keeps the theoretical question clear: whether employees who regard work as a domain for growth, challenge, competence, and meaningful achievement are more likely to report proactive behavior, and whether this association depends on contextual support and personal agency. A focused model also avoids diluting the theoretical logic across value domains that may imply different behavioral mechanisms.

The hypotheses are therefore parsimonious but theoretically layered. H1 examines the value-behavior link by asking whether intrinsic work values are associated with proactive behavior. H2 examines the contextual boundary condition by asking whether perceived employment relations climate changes the strength of that association. H3 examines the dispositional boundary condition by asking whether internal locus of control strengthens value enactment. Together, the three hypotheses move the manuscript beyond a simple antecedent-outcome design. They position proactive behavior as the result of motivational content, situational meaning, and agency belief operating together.

**Figure 1:**
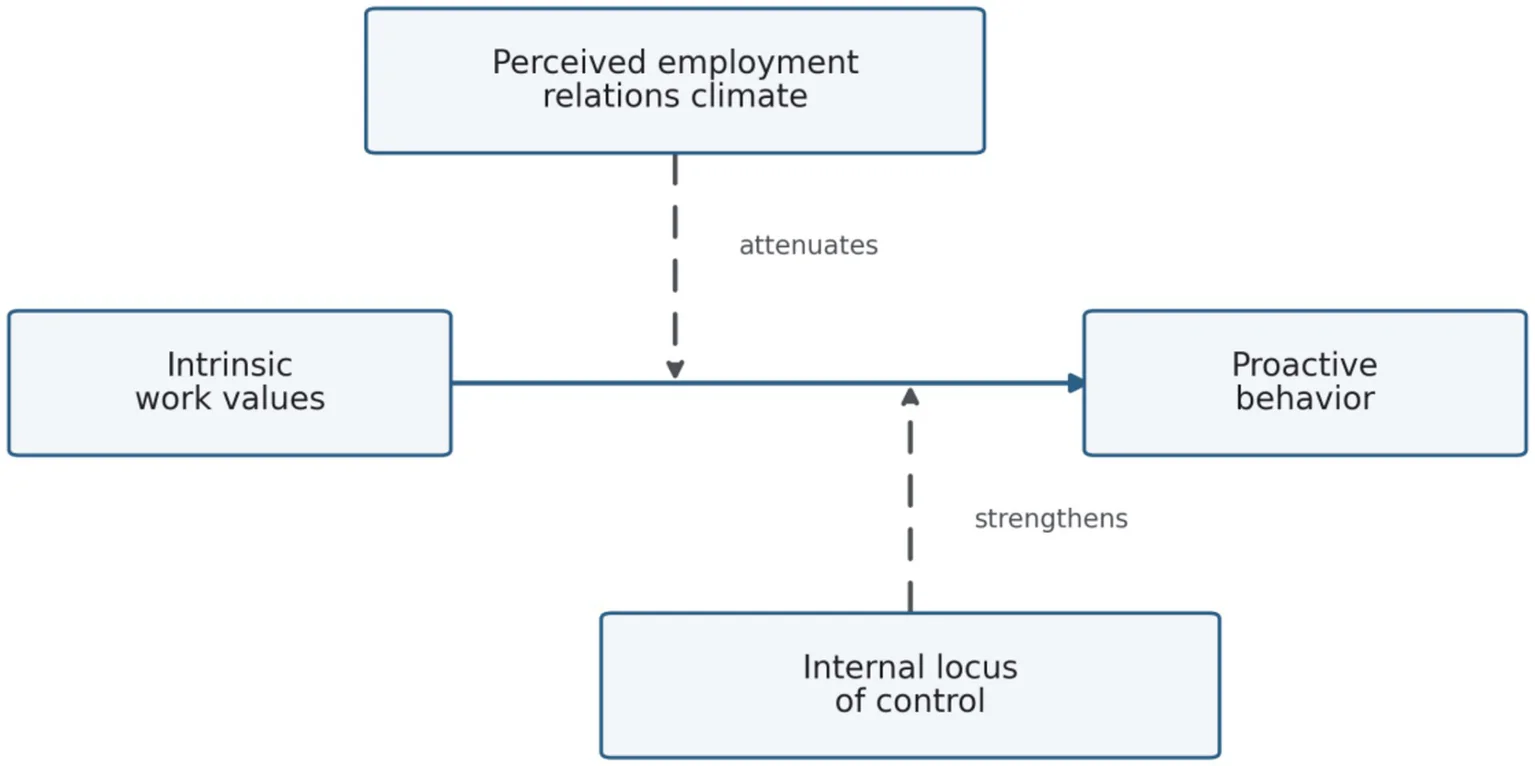
Conceptual model. The solid arrow denotes the focal association; dashed arrows terminate directly at two distinct points on the focal path and denote moderation.

Figure 1 summarizes the proposed model. Intrinsic work values are positioned as the focal motivational antecedent of proactive behavior. Perceived employment relations climate and internal locus of control are positioned as two complementary boundary conditions: one external and contextual, the other internal and dispositional. This structure allows the study to explain not only whether intrinsic work values matter for early-career proactivity, but also when those values are most likely to be expressed in proactive behavior.

## 3 Materials and methods

### 3.1 Participants and procedure

The study targeted early-career employees, operationally defined as individuals whose cumulative full-time work experience did not exceed 24 months. This threshold was chosen to capture an early role-learning period rather than organizational tenure in a single employer: respondents could be labor-market entrants or employees in their first 2 years of cumulative work experience. The 24-month cutoff was adopted as a study-specific sampling convention rather than as a universal developmental boundary. This operationalization allowed the study to focus on employees in the early phase of organizational entry, during which role learning, adjustment, and the formation of workplace expectations remain particularly salient (Van Maanen and Schein, 1979; Kammeyer-Mueller and Wanberg, 2003; Saks et al., 2007). Participants were recruited through the Wenjuanxing online questionnaire platform and completed the survey electronically in Chinese. The questionnaire included measures of intrinsic work values, proactive behavior, perceived employment relations climate, internal locus of control, and demographic information. To ensure data quality, the following procedures were applied: (a) Wenjuanxing was configured to permit only one submission per IP/device identifier; responses were excluded if participants (b) did not meet the employment eligibility criterion, including being unemployed or reporting more than 24 months of cumulative full-time work experience; (c) failed an embedded attention-check item; (d) completed any major scale at an average response time of less than 1 second per item. Of 605 returned questionnaires, 64 were excluded and 541 valid responses were retained for analysis, yielding a valid-response retention rate among returned questionnaires of 89.4%. Participants received a cash incentive of RMB 5 through Wenjuanxing upon completion of the questionnaire. Completion time was available as an additional aggregate diagnostic: the archived valid survey records had a median completion time of 301 s (interquartile range = 206.5–400.0 s).

Participation was anonymous and voluntary. All procedures were approved by the Ethics Committee of Southwest University, and informed consent was obtained from all participants prior to survey administration. The study was conducted in accordance with the ethical principles of the Declaration of Helsinki, and all data were collected anonymously in compliance with institutional data protection standards.

The final analytic sample included 271 men (50.1%) and 270 women (49.9%). Regarding relationship status, 238 participants were single (44.0%), 177 were in a relationship (32.7%), and 126 were married (23.3%). Regarding educational attainment, 288 participants held a bachelor’s degree (53.2%), 143 held a master’s degree (26.4%), 69 had an associate degree or below (12.8%), and 41 held a doctoral degree (7.6%). Monthly income ranged from below 2,000 RMB to above 10,000 RMB, with the largest segment earning 4,000–6,000 RMB (28.1%), followed by 6,000–10,000 RMB (25.1%) and 2,000–4,000 RMB (18.3%). Participants worked in public institutions (32.5%), private enterprises (30.7%), state-owned or government organizations (16.6%), foreign or joint ventures (9.8%), and other organization types (10.4%). Work experience ranged from 0 to 24 months in the retained sample, with 35.5% reporting 0–6 months, 17.6% reporting 7–12 months, 21.1% reporting 13–18 months, and 25.9% reporting 19–24 months. Participant demographic characteristics are presented in Table 1.

**Table 1:** Socio-demographic information of participants (*n* = 541).

Variable	Category	*n*	%	Variable	Category	*n*	%
Gender	Male	271	50.1	Academic background	Science/Engineering	200	37.0
	Female	270	49.9		Humanities/Social sciences	197	36.4
Relationship status	Single	238	44.0		Arts	60	11.1
	In a relationship	177	32.7		Medicine	47	8.7
	Married	126	23.3		Other	37	6.8
Work experience	0–6 months	192	35.5	Monthly income	RMB 4,000–6,000	152	28.1
	19–24 months	140	25.9		RMB 6,000-10,000	136	25.1
	13–18 months	114	21.1		RMB 2,000-4,000	99	18.3
	7–12 months	95	17.6		>RMB 10,000	78	14.4
Education	Bachelor	288	53.2		<RMB 2,000	76	14.0
	Master	143	26.4	Organization type	Public institution	176	32.5
	Associate degree or below	69	12.8		Private enterprise	166	30.7
	Doctorate	41	7.6		State-owned/government	90	16.6
Household registration	Rural	278	51.4		Other	56	10.4
	Urban	263	48.6		Foreign/joint venture	53	9.8

### 3.2 Measures

#### 3.2.1 Demographic information

Demographic information was collected using several items asking participants about their gender, relationship status, academic background, educational attainment, average monthly income, years of employment, organization type, and household registration. Years of employment was used to confirm the early-career inclusion criterion. For hypothesis testing, all demographic variables reported in Table 1 were entered as covariates in the first block of the hierarchical regression models. Categorical variables were dummy-coded, and the complete covariate coefficients are reported in Table 2.

**Table 2:** Covariate coefficients in the final hierarchical regression model predicting proactive behavior.

Covariate	*B*	Robust SE	*t*	*p*
Gender (male vs. female)	1.024	1.034	0.991	0.322
Relationship status (married vs. in a relationship)	0.714	1.470	0.486	0.627
Relationship status (single vs. in a relationship)	−0.631	0.868	−0.726	0.468
Academic background (humanities/social sciences vs. arts)	−1.012	2.001	−0.506	0.613
Academic background (medicine vs. arts)	0.755	2.490	0.303	0.762
Academic background (other vs. arts)	−1.323	2.077	−0.637	0.525
Academic background (science/engineering vs. arts)	−2.285	1.987	−1.150	0.251
Education (bachelor vs. associate degree or below)	1.062	1.630	0.652	0.515
Education (doctorate vs. associate degree or below)	3.618	2.455	1.473	0.141
Education (master vs. associate degree or below)	3.155	1.737	1.817	0.070
Monthly income (RMB 4,000–6,000 vs. RMB 2,000–4,000)	0.314	1.269	0.247	0.805
Monthly income (RMB 6,000–10,000 vs. RMB 2,000–4,000)	1.694	1.328	1.275	0.203
Monthly income (<RMB 2,000 vs. RMB 2,000–4,000)	−1.445	1.397	−1.034	0.302
Monthly income (>RMB 10,000 vs. RMB 2,000–4,000)	3.087	1.724	1.790	0.074
Work experience (13–18 months vs. 0–6 months)	−1.092	1.322	−0.826	0.409
Work experience (19–24 months vs. 0–6 months)	−0.264	1.180	−0.224	0.823
Work experience (7–12 months vs. 0–6 months)	1.233	1.386	0.890	0.374
Organization type (other vs. foreign/joint venture)	−1.410	1.871	−0.754	0.451
Organization type (private enterprise vs. foreign/joint venture)	−0.965	1.653	−0.584	0.559
Organization type (public institution vs. foreign/joint venture)	−1.942	1.780	−1.091	0.276
Organization type (state-owned/government vs. foreign/joint venture)	−1.208	1.902	−0.635	0.525
Household registration (urban vs. rural)	−1.386	0.827	−1.675	0.095

Coefficients are from the final model (M4), which included intrinsic work values, perceived employment relations climate, internal locus of control, and the two interaction terms. Categorical covariates were dummy-coded; reference categories are embedded in the covariate labels. HC3 robust standard errors are reported. *p < 0.05; **p < 0.01; ***p < 0.001.

#### 3.2.2 Intrinsic work values scale

The Intrinsic Work Values Scale was used to measure employees’ self-reported intrinsic work values (Chen, 2012). It is an 11-item unidimensional scale assessing the extent to which employees value growth, learning, challenge, self-development, and meaningful accomplishment at work, with a representative item being “Work gives me opportunities to learn and promotes my personal development.” Responses were recorded on a six-point importance scale ranging from 1 (very unimportant) to 6 (very important). Higher scores indicated stronger intrinsic work values. In the current study, Cronbach’s alpha was 0.96.

#### 3.2.3 Proactive behavior scale

The Proactive Behavior Scale was used to measure employees’ self-reported proactive behavior at work (Frese et al., 1997; Li, 2011). It is a 12-item scale with three dimensions: self-starting behavior (5 items), future-oriented action (4 items), and persistence in overcoming obstacles (3 items), with a representative item being “I actively solve problems.” Items were rated on a five-point agreement scale ranging from 1 (very inconsistent with me) to 5 (very consistent with me). Reverse-coded items were recoded following the questionnaire scoring instructions so that higher scores represented stronger proactive behavior. Cronbach’s alpha was 0.94 in this study.

#### 3.2.4 Perceived employment relations climate scale

The Perceived Employment Relations Climate Scale was used to measure employees’ individual perceptions of employment relations climate (Chen et al., 2013; Fan L. et al., 2022; Fan X. et al., 2022). The six administered items were drawn from Chinese labor-management relationship climate research and assessed perceived cooperation, mutual respect, promise keeping, perspective taking, negotiation integrity, and fairness in labor-management relations, with a representative item being “At work, managers and I work together to make the work environment better.” Responses were recorded on a seven-point agreement scale ranging from 1 (strongly disagree) to 7 (strongly agree). Higher scores indicated a more favorable perceived employment relations climate. Because respondents were sampled individually and no team- or organization-level aggregation was available, the construct is interpreted as an individual-level perceived contextual cue rather than as an aggregated unit-level climate. Cronbach’s alpha was 0.96.

#### 3.2.5 Internal locus of control scale

The Internal Locus of Control Scale was used to measure internal locus of control in work-related contexts (Spector, 1988; Xi, 2015). It is a 14-item scale containing internal-control and external-control statements, with a representative item being “Job opportunities are created by oneself.” Items were rated on a five-point agreement scale ranging from 1 (very inconsistent with me) to 5 (very consistent with me). External-control items were reverse-scored so that higher scores indicated stronger internal locus of control. In the current study, Cronbach’s alpha for the full scale was 0.67. Because the instrument combines directly worded internal-control statements with reverse-keyed external-control statements, its internal consistency is susceptible to item-polarity and wording effects even after scoring directions are aligned.

### 3.3 Data analysis

Data analysis proceeded in five steps. First, internal consistency coefficients, descriptive statistics, Pearson correlations, and common method bias diagnostics were computed. Second, confirmatory factor analysis was conducted in Mplus 8.3 using maximum-likelihood estimation to evaluate the hypothesized four-factor measurement structure. Model fit was assessed using chi-square, the comparative fit index (CFI), the Tucker-Lewis index (TLI), the root mean square error of approximation (RMSEA), and the standardized root mean square residual (SRMR). Modification indices, standardized residuals, and item content were reviewed jointly to identify localized dependence attributable to closely overlapping content, parallel wording, or shared reverse-keying within the same construct. The measurement model freely estimated nine such within-construct residual covariances; all cross-loadings and cross-construct residual covariances remained fixed to zero. Measurement evidence also included standardized item loadings, composite reliability, average variance extracted, the Fornell-Larcker criterion, and HTMT ratios (Fornell and Larcker, 1981; Henseler et al., 2015; Hu and Bentler, 1999; Brown, 2015; Hair et al., 2019; Kline, 2023). Third, the hypotheses were tested using hierarchical regression models predicting proactive behavior. All demographic variables reported in Table 1 were dummy-coded and entered as covariates in the first block of each model; their coefficients and significance levels in the final model are reported in Table 2. Continuous predictors and moderators were mean-centered before interaction terms were created (Aiken and West, 1991; Cohen et al., 2003). Fourth, significant interactions were probed using simple-slope analyses, Johnson-Neyman regions of significance, and interaction plots with 95% confidence bands (Preacher et al., 2006; Hayes, 2022). Fifth, robustness checks examined whether adding intrinsic work values by work-experience-band interactions improved model fit. HC3 robust standard errors were used to reduce sensitivity to heteroskedasticity, and two-tailed significance tests were evaluated at *p* < 0.05.

## 4 Results

### 4.1 Measurement model and common method bias

Before testing the hypotheses, confirmatory factor analysis was conducted to evaluate whether intrinsic work values, proactive behavior, perceived employment relations climate, and internal locus of control were empirically distinguishable. The four-factor model included nine within-construct residual covariances supported jointly by modification indices and item content: PB1 with PB2 (immediate identification and resolution of work problems), PB11 with PB12 (future-oriented work and career planning), PERC1 with PERC2 (cooperative and respectful employment relations), PERC5 with PERC6 (integrity and fairness of employment agreements), LOC1 with LOC3 (personal-agency beliefs), LOC2 with LOC6 (effort and generalized work-competence beliefs), LOC10 with LOC13 (performance-contingent advancement and reward), LOC5 with LOC14 (reverse-keyed attribution of remuneration to luck), and LOC12 with LOC14 (reverse-keyed attribution of work outcomes to luck). Factor cross-loadings and residual covariances between indicators of different constructs were fixed to zero.

The four-factor model yielded *χ*^2^ (845) = 2418.43, CFI = 0.922, TLI = 0.917, RMSEA = 0.059, 90% CI [0.056, 0.061], and SRMR = 0.098. Relative to the corresponding four-factor specification in which indicator residual covariances were fixed to zero, *χ*^2^ (854) = 2911.19, CFI = 0.899, TLI = 0.893, RMSEA = 0.067, and SRMR = 0.099, the nested-model comparison was significant, Δ*χ*^2^ (9) = 492.76, *p* < 0.001. All nine residual covariances were statistically significant (*p* < 0.001). The absolute change in each latent-construct correlation was no greater than 0.011 across the two specifications, indicating that the factor relationships remained stable. The four-factor model also fit substantially better than the three-factor and one-factor alternatives reported in Table 3, supporting the empirical distinctiveness of the four focal constructs.

**Table 3:** Measurement model fit comparisons.

Model	*χ*^2^ (d*f*)	CFI	TLI	RMSEA	SRMR
Four-factor model	2418.43 (845)	0.922	0.917	0.059	0.098
Four-factor model (residual covariances fixed to zero)	2911.19 (854)	0.899	0.893	0.067	0.099
Three-factor model (IWV + LOC combined)	7440.42 (857)	0.676	0.658	0.119	0.180
One-factor model	14490.26 (860)	0.329	0.295	0.171	0.237

Models were estimated in Mplus 8.3 using maximum-likelihood estimation. The four-factor model included the nine theoretically supported within-construct residual covariances described in Section 4.1; the more restrictive four-factor specification fixed these residual covariances to zero. No cross-loadings or cross-construct residual covariances were estimated. The nested-model comparison yielded Δ*χ*^2^ (9) = 492.76, *p* < 0.001. CFI, comparative fit index; TLI, Tucker-Lewis index; RMSEA, root mean square error of approximation; SRMR, standardized root mean square residual. The three-factor model combined intrinsic work values and internal locus of control.

Convergent validity was evaluated using standardized item loadings, composite reliability, and average variance extracted. All standardized loadings were statistically significant (*p* < 0.001) and ranged from 0.46 to 0.93 in absolute magnitude. Composite reliability estimates ranged from 0.93 to 0.96, and AVE estimates ranged from 0.52 to 0.80. The square roots of AVE ranged from 0.72 to 0.89 and exceeded the largest absolute latent-construct correlation (|*r*| = 0.56). HTMT ratios ranged from 0.18 to 0.54, below the commonly used 0.85 criterion, providing additional evidence of discriminant validity. The standardized item loadings and construct-level reliability and validity estimates are reported in Table 4. Cronbach’s alpha for internal locus of control was 0.67; this coefficient is reported alongside its composite reliability and AVE to provide a complete account of the measurement evidence.

**Table 4:** Standardized CFA item loadings and construct validity diagnostics.

Construct	Item	Standardized loading
Intrinsic work values	IWV1	0.872
	IWV2	0.693
	IWV3	0.866
	IWV4	0.862
	IWV5	0.886
	IWV6	0.849
	IWV7	0.751
	IWV8	0.822
	IWV9	0.711
	IWV10	0.856
	IWV11	0.832
Proactive behavior	PB1	0.779
	PB2	0.773
	PB3	0.801
	PB4	0.791
	PB5	0.835
	PB6	0.766
	PB7	0.861
	PB8	0.818
	PB9	0.502
	PB10	0.770
	PB11	0.768
	PB12	0.762
Perceived employment relations climate	PERC1	0.862
	PERC2	0.907
	PERC3	0.930
	PERC4	0.924
	PERC5	0.902
	PERC6	0.828
Internal locus of control	LOC1	0.456
	LOC2	0.534
	LOC3	0.515
	LOC4	0.784
	LOC5	0.874
	LOC6	0.577
	LOC7	0.894
	LOC8	0.860
	LOC9	0.745
	LOC10	0.494
	LOC11	0.844
	LOC12	0.811
	LOC13	0.544
	LOC14	0.860

Loadings are standardized (STDYX) coefficients from the four-factor CFA estimated in Mplus 8.3. For locus-of-control indicators, external-control items were reverse-scored and loading magnitudes are presented in absolute terms. All standardized loadings were statistically significant at *p* < 0.001. Alpha/CR/AVE values were as follows: intrinsic work values = 0.96/0.96/0.67; proactive behavior = 0.94/0.95/0.60; perceived employment relations climate = 0.96/0.96/0.80; internal locus of control = 0.67/0.93/0.52.

To examine potential common method bias, exploratory statistical procedures were conducted following established recommendations (Podsakoff et al., 2003; Richardson et al., 2009; Fuller et al., 2016). First, Harman’s single-factor test was performed using unrotated principal-components analysis of the focal measurement items (Harman, 1967). The results showed that the first unrotated factor accounted for 26.12% of the total variance, which was well below the conservative 40% threshold. Moreover, five factors had eigenvalues greater than 1, indicating that the covariance structure was not dominated by a single general factor. This diagnostic was interpreted alongside the CFA comparison rather than as a standalone remedy, consistent with recent cautions about common-method assessment in survey research (Baumgartner and Weijters, 2021; Manata and Boster, 2024).

As an auxiliary diagnostic, education level and organization type were examined in relation to the focal constructs. Dummy-coded correlations between education categories and the focal variables ranged from |*r*| = 0.01 to 0.25, and dummy-coded correlations involving organization type ranged from |*r*| = 0.01 to 0.20. These coefficients indicate small to modest background-variable associations rather than a dominant common source, and the corresponding demographic variables were retained as controls in the hypothesis tests. Taken together, the exploratory factor results and covariate-adjusted modeling strategy suggest that common method bias was not a serious concern in the present analyses.

### 4.2 Descriptive statistics and correlations

Correlation analysis was conducted to examine the bivariate relationships among intrinsic work values, proactive behavior, perceived employment relations climate, and internal locus of control (see Table 5). Intrinsic work values were positively correlated with proactive behavior (*r* = 0.13, *p* < 0.01), providing preliminary support for the proposed value-behavior link. Perceived employment relations climate was also positively correlated with proactive behavior (*r* = 0.19, *p* < 0.001), suggesting that employees who perceived more favorable labor-management relations tended to report higher proactive behavior. Internal locus of control was not significantly correlated with proactive behavior at the bivariate level (*r* = −0.07, *p* = 0.083), which is consistent with the theoretical expectation that its role is better understood as a boundary condition rather than as a simple main-effect predictor.

**Table 5:** Descriptive statistics, reliabilities, and correlations among focal variables.

Variables	*M*	SD	1	2	3	4
1. Intrinsic work values	48.06	11.32	(0.96)			
2. Proactive behavior	44.42	9.77	0.13**	(0.94)		
3. Perceived employment relations climate	28.77	7.71	0.52***	0.19***	(0.96)	
4. Internal locus of control	45.82	7.14	0.34***	−0.07	0.42***	(0.67)

*N* = 541. Cronbach’s alpha coefficients are shown in parentheses on the diagonal. *p < 0.05; **p < 0.01; ***p < 0.001.

The predictors and moderators were moderately interrelated. Intrinsic work values were positively associated with perceived employment relations climate (*r* = 0.52, *p* < 0.001) and internal locus of control (*r* = 0.34, *p* < 0.001), while perceived employment relations climate was positively associated with internal locus of control (*r* = 0.42, *p* < 0.001). These correlations indicate meaningful overlap among value orientation, perceived relational climate, and agency beliefs, but they do not suggest redundancy among the focal constructs. The correlation results therefore provide an initial empirical basis for the regression and moderation analyses reported below.

### 4.3 Hypothesis tests

Table 6 summarizes the hierarchical regression models. Demographic covariates were entered first as a block (M1), intrinsic work values were then added to test the value-behavior association (M2), perceived employment relations climate and internal locus of control were added in M3, and the two interaction terms were added in M4. This stepwise presentation separates the incremental contribution of intrinsic work values from the conditional associations tested in H2 and H3. The complete covariate coefficients from the final model are reported in Table 2.

**Table 6:** Hierarchical regression models predicting proactive behavior.

Model	Terms added	IWV	PERC	LOC	IWV × PERC	IWV × LOC	*R* ^2^	Δ*R*^2^	Δ*F*
M1	Demographic controls						0.064	0.064	1.60*
M2	+Intrinsic work values	0.179 (0.057)**					0.099	0.035	20.01***
M3	+Perceived employment relations climate and internal locus of control	0.087 (0.055)	0.354 (0.084)***	−0.115 (0.067)			0.147	0.048	14.45***
M4	+Two interaction terms	0.081 (0.049)	0.338 (0.083)***	−0.169 (0.072)*	−0.017 (0.005)**	0.021 (0.007)**	0.180	0.033	10.32***

IWV, intrinsic work values; PERC, perceived employment relations climate; LOC, internal locus of control. Entries for focal predictors are unstandardized coefficients with HC3 robust standard errors in parentheses. *p < 0.05; **p < 0.01; ***p < 0.001.

H1 was supported. After demographic controls were entered, intrinsic work values explained additional variance in proactive behavior (Delta *R*^2^ = 0.035, Delta *F* = 20.01, *p* < 0.001) and were positively associated with proactive behavior (*B* = 0.179, SE = 0.057, *p* = 0.002). The full interaction model explained 18.0% of the variance in proactive behavior. As shown in Table 2, the demographic block explained a small but statistically significant amount of variance when entered first (M1: *R*^2^ = 0.064, Delta *F* = 1.60, *p* = 0.041). In the final covariate-adjusted model, however, no individual demographic dummy coefficient reached the 0.05 significance level.

H2 was supported. In the full model, the interaction between intrinsic work values and perceived employment relations climate was negative and significant (*B* = −0.017, SE = 0.005, *p* = 0.002). This pattern is interpreted as an attenuation effect consistent with strong-situation reasoning, not as evidence that a favorable perceived climate is harmful. H3 was also supported: the interaction between intrinsic work values and internal locus of control was positive and significant (*B* = 0.021, SE = 0.007, *p* = 0.001). The block of interaction terms added significant explanatory power beyond the main-effects model (Delta *R*^2^ = 0.033, Delta *F* = 10.32, *p* < 0.001).

Multicollinearity diagnostics did not indicate problematic overlap among the focal predictors. VIF values for intrinsic work values, perceived employment relations climate, internal locus of control, and the two interaction terms ranged from 1.57 to 1.87. The maximum VIF across the full model was 5.27 and was associated with a dummy-coded demographic category rather than with a focal theoretical variable. A robustness check adding interactions between intrinsic work values and work-experience bands did not significantly improve model fit (Delta *R*^2^ = 0.010, *F*(3, 510) = 2.11, *p* = 0.098), suggesting that the focal value-behavior association was not driven solely by a particular tenure band within the 0–24 month early-career range (see Table 7).

**Table 7:** Simple slopes for intrinsic work values.

Interaction	Moderator level	Slope *B*	SE	*t*	*p*
Intrinsic × perceived climate	Low (−1 SD)	0.210	0.061	3.43***	0.001
Intrinsic × perceived climate	Mean	0.081	0.049	1.66	0.098
Intrinsic × perceived climate	High (+1 SD)	−0.047	0.066	−0.71	0.478
Intrinsic × internal LOC	Low (−1 SD)	−0.068	0.064	−1.08	0.282
Intrinsic × internal LOC	Mean	0.081	0.049	1.66	0.098
Intrinsic × internal LOC	High (+1 SD)	0.231	0.072	3.23**	0.001

Slopes represent the association between intrinsic work values and proactive behavior.

Johnson-Neyman probing further clarified the regions of significance. For perceived employment relations climate, the positive association between intrinsic work values and proactive behavior was significant when climate scores were below 27.89 on the observed sum-score scale; above this point, the slope was not statistically different from zero within the observed range. For internal locus of control, the value-behavior association became significantly positive when internal locus of control exceeded 46.62. The lower-locus-of-control simple slope was negative in direction but nonsignificant and should not be interpreted as evidence that intrinsic work values reduce proactive behavior. Rather, the pattern indicates that intrinsic work values were reliably associated with proactive behavior only when employees reported stronger internal control beliefs.

**Figure 2:**
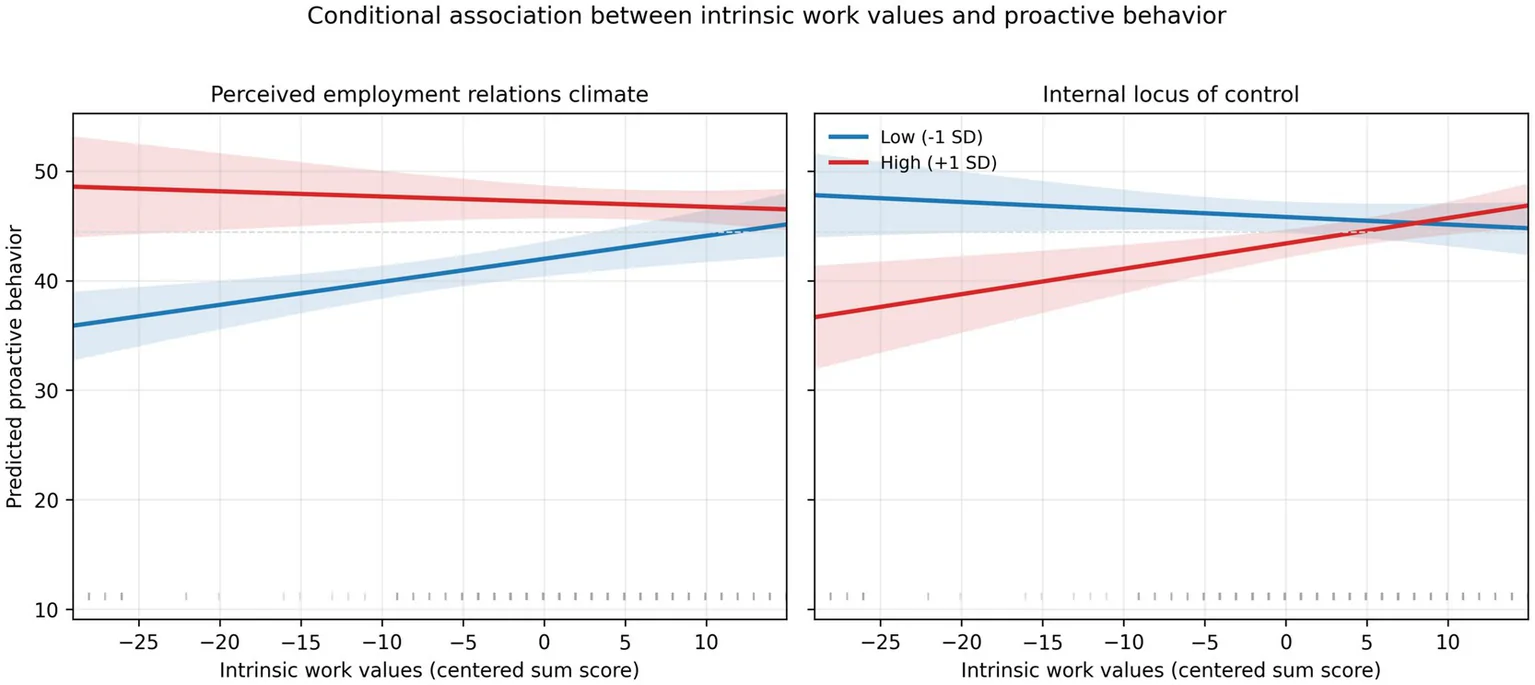
Interaction plots for intrinsic work values. Lines are based on the full centered regression equation, with covariates held at their sample means. Shaded areas represent 95% confidence bands; rug marks show the observed distribution of intrinsic work values. The horizontal axes report centered intrinsic work values, and the vertical axis reports predicted proactive behavior.

Figure 2 shows how the association between intrinsic work values and proactive behavior varies across moderator levels. In the perceived employment relations climate panel, the low-climate line has a positive slope, whereas the high-climate line is comparatively flat, consistent with an attenuating effect. In the internal locus of control panel, the high-control line has a positive slope, whereas the low-control line is not statistically different from zero.

## 5 Discussion

This study examined whether intrinsic work values are associated with proactive behavior among early-career employees and whether this association depends on perceived employment relations climate and internal locus of control. The findings support a conditional value-based account. Intrinsic work values were positively related to proactive behavior in the main-effect model, but the strength of this association varied across contextual and dispositional conditions. The association was stronger when perceived employment relations climate was lower and when internal locus of control was higher. These patterns suggest that growth-oriented work values matter for early-career proactivity, but they are most behaviorally consequential when employees need to rely on their own values and when they believe their actions can influence work outcomes.

### 5.1 Theoretical and practical implications

The present findings contribute to proactive behavior research by clarifying a value-based route to early-career initiative. Proactivity has usually been explained through proactive personality, role-breadth self-efficacy, job autonomy, leadership, voice opportunities, future work selves, or socialization tactics (Bateman and Crant, 1993; Parker et al., 2006; Grant and Ashford, 2008; Fuller and Marler, 2009; Parker et al., 2010; Strauss et al., 2012; Morrison, 2023). These accounts explain whether employees are inclined, enabled, or invited to act. The present study adds a complementary motivational explanation: early-career employees may behave proactively because they construe work itself as a domain for learning, challenge, competence development, and meaningful accomplishment. This extension matters because proactive behavior is discretionary, uncertain, and sometimes politically delicate; employees need not only opportunity but also a personally legitimate reason to invest in action that exceeds formal requirements (Crant, 2000; Parker and Collins, 2010; Wang et al., 2025).

The emphasis on intrinsic work values is central rather than cosmetic. We do not argue that work values as a broad omnibus construct, or a general Chinese work-values profile, should predict proactive behavior in the same way. Values research has long treated work values as evaluative standards that guide occupational choice, work adjustment, and job attitudes (Super, 1962; Dawis and Lofquist, 1984; Arieli et al., 2020; Chang et al., 2024). The present findings suggest a more specific claim: the intrinsic dimension of work values is especially relevant to proactive behavior because its content is developmental rather than merely instrumental. Pay, security, status, interpersonal harmony, and leisure-oriented values may be important for career choice and well-being, but they do not necessarily make self-initiated change meaningful. Intrinsic work values, by contrast, define work as a site for growth, mastery, challenge, achievement, autonomy, and meaningful self-development (Chen, 2012). These contents map closely onto what proactive behavior requires: learning from feedback, experimenting with improvements, tolerating uncertainty, and investing effort before immediate rewards are available.

This construct-specific interpretation also refines the values-behavior literature. Classic values-behavior theory argues that values are more likely to guide action when the behavior is relevant to the value and when the situation permits behavioral expression (Bardi and Schwartz, 2003; Schwartz, 2012). Proactive behavior is a particularly appropriate behavioral domain for testing this logic because it contains substantial discretion: employees must decide whether to seek information, voice suggestions, prevent problems, or improve work methods before being asked. By showing that intrinsic work values are associated with proactive behavior, the study moves work-values research beyond evaluative outcomes such as satisfaction, commitment, person-organization fit, or turnover intention. This extension complements person-organization fit research, which has long shown that value congruence matters for attitudes and adjustment but has less often addressed self-initiated change behavior (Chatman, 1989; Kristof-Brown et al., 2005). It suggests that intrinsic values may function not only as standards for judging whether work is desirable, but also as standards for deciding whether one should actively shape work.

The study also speaks to proactive motivation theory. Parker et al. (2010) proposed that proactive behavior depends on reasons to act, perceived capability to act, and energized states. Much subsequent research has emphasized capability and opportunity, including autonomy, role breadth, mentoring, and supportive leadership (Parker et al., 2006; Griffin et al., 2007; Yang et al., 2024). Intrinsic work values help specify the ‘reason to’ component. They give proactive behavior developmental meaning: feedback seeking becomes an avenue for learning, problem solving becomes an opportunity for mastery, and improvement-oriented effort becomes part of constructing a competent work identity. This is related to self-determination theory but not reducible to intrinsic motivation. Intrinsic motivation refers to the experienced enjoyment of an activity, whereas intrinsic work values refer to stable beliefs that work should provide growth, challenge, and self-realization (Deci and Ryan, 2000; Gagne and Deci, 2005).

The early-career setting sharpens this contribution. Organizational socialization research has shown that newcomers are active participants in their own adjustment, using information seeking, feedback seeking, and relationship building to reduce uncertainty and develop role clarity (Van Maanen and Schein, 1979; Morrison, 1993; Bauer et al., 2007; Saks et al., 2007). Recent meta-analytic work likewise indicates that newcomer proactive behaviors are generally beneficial for socialization outcomes (Zhao et al., 2023). The present study extends this literature by identifying a value content that may make such proactive behavior personally meaningful. Early-career employees high in intrinsic work values are not merely trying to adapt; they may be using proactive behavior to enact a desired professional self organized around learning, competence, and meaningful contribution.

Practically, this means that onboarding should not reduce proactivity to a vague exhortation to ‘take initiative.’ Many organizations ask newcomers to be proactive but provide little guidance about what kinds of initiative are appropriate, which issues are safe to raise, or how proactive effort connects to development. A value-sensitive onboarding process would help newcomers articulate learning goals, identify growth-oriented assignments, and connect early proactive behaviors to competence development. This recommendation is consistent with socialization research showing that adjustment depends on both organizational tactics and newcomer agency (Bauer et al., 2007; Saks et al., 2007), and with recent evidence that mentoring can support new employees’ proactive behavior (Yang et al., 2024; Lee, 2024).

The moderating effect of perceived employment relations climate provides a second theoretical contribution by qualifying a simple support-enhancement account. At first glance, one might expect favorable relational climates always to strengthen the effect of intrinsic work values. The present pattern suggests a more nuanced strong-situation interpretation. When employees perceive labor-management relations as cooperative, respectful, and fair, proactive behavior may become more normatively supported and less dependent on individual differences in intrinsic work values. A positive perceived climate can therefore raise the general legitimacy of initiative while reducing the extent to which only highly intrinsically oriented employees act proactively. This is not a claim that favorable climate weakens employees in a harmful sense; it is a claim about reduced between-person variability under clearer contextual cues.

This interpretation also connects the present findings to recent voice and proactivity research. Studies of employee voice show that managers’ responses, hierarchy, and mixed signals can determine whether employees speak up or remain silent (Burris et al., 2022; Parke et al., 2022; Morrison, 2023). The present results suggest that perceived employment relations climate may operate as a background signal that shapes the behavioral relevance of individual values. A favorable climate does not make intrinsic work values unimportant; rather, it may make proactive behavior less exclusively dependent on those values by making initiative psychologically safer and socially more intelligible for a wider range of employees. This distinction prevents the misleading interpretation that positive climate is harmful. The pattern is an attenuation or compensatory effect, not an adverse effect of good labor-management relations.

For practice, managers should treat perceived employment relations climate as a platform that democratizes proactive behavior. If initiative is only shown by highly intrinsically oriented employees, the organization may be relying too heavily on individual self-drive. Respectful communication, transparent follow-up on suggestions, fair conflict-resolution procedures, and visible recognition of constructive initiative can reduce ambiguity about whether proactive behavior is welcome. These practices are particularly important in early-career contexts, where employees often lack status and may be uncertain about the interpersonal risks of challenging routines. When relational climates are weak, supervisors should provide additional protection, explicit permission to raise concerns, and low-risk channels for suggestions so that intrinsic work values can be expressed without excessive psychological cost.

The moderating role of internal locus of control provides a third theoretical contribution by clarifying the agency condition under which intrinsic work values are associated with proactive behavior. Values identify what employees consider important, but they do not guarantee that employees view action as consequential. The finding that intrinsic work values were linked to proactive behavior mainly under higher internal locus of control suggests that growth-oriented values are more likely to matter behaviorally when employees believe their own actions can influence work outcomes. This interpretation positions locus of control as an enactment condition rather than as a substitute for intrinsic values or merely as another positive trait.

The interaction with internal locus of control is consistent with trait activation theory. Trait-like dispositions should matter most when situations contain cues that make the disposition relevant (Tett and Burnett, 2003). Proactive behavior contains exactly such cues because it requires personal responsibility, action under uncertainty, and the belief that effort can influence future states. Early-career employees may endorse intrinsic work values, yet still refrain from initiative if they assume that outcomes are controlled by supervisors, hierarchy, chance, or opaque organizational rules. When internal locus of control is higher, intrinsic values have a stronger pathway to behavior because employees are more likely to interpret proactive opportunities as controllable situations rather than futile or risky attempts.

This agency interpretation has an important theoretical implication. It suggests that intrinsic work values should not be treated as self-executing motivational forces. Employees may value learning, challenge, and meaningful achievement, but those values are more likely to become proactive behavior when employees also believe that their own actions can shape work outcomes. This view is consistent with Galvin et al.’s (2018) argument that locus of control is most useful in organizational research when it is tied to specific agentic processes rather than treated as a generic background trait. In the present model, internal locus of control helps explain when growth-oriented values are translated into action: it supplies the perceived controllability needed for employees to invest effort in uncertain, self-starting, and future-oriented behavior.

Practically, organizations can strengthen this agency condition through structured mastery experiences. Newcomers with lower internal control beliefs may benefit from bounded improvement projects, rapid feedback, visible supervisor responsiveness, and mentoring that helps them interpret setbacks as information rather than as evidence that initiative is useless. These practices are consistent with self-determination theory’s emphasis on competence support and autonomy-supportive contexts (Deci and Ryan, 2000; Gagne and Deci, 2005), and with newcomer socialization research showing that support and guidance can facilitate proactive adjustment (Bauer et al., 2007; Yang et al., 2024). The aim is not to change personality directly, but to create repeated experiences of controllable progress through which intrinsic work values become behaviorally actionable.

Taken together, the findings caution against both over-individualized and over-contextualized accounts of early-career proactivity. An over-individualized view would imply that organizations merely need to recruit intrinsically motivated newcomers; an over-contextualized view would imply that climate alone is sufficient. The present results support a person-context interpretation: intrinsic work values provide developmental meaning, perceived employment relations climate provides social information about the legitimacy of initiative, and internal locus of control provides perceived agency. This integrated account is consistent with broader arguments that context is essential for understanding organizational behavior (Johns, 2006) and with contemporary proactivity scholarship emphasizing wise, context-sensitive initiative rather than indiscriminate action (Wang et al., 2025).

### 5.2 Limitations and future directions

First, the design was cross-sectional and self-reported, so causal inference is not warranted. Although the theoretical model treats intrinsic work values as an antecedent-like value orientation, proactive work experiences may also strengthen intrinsic work values over time. Future research should use longitudinal, diary, experimental, or multi-source designs to examine temporal ordering and reduce common-source concerns. Second, perceived employment relations climate was measured at the individual level. The present study therefore speaks to perceived contextual cues rather than aggregated team- or organization-level climate. Future studies should collect nested data and examine whether unit-level climate, individual climate perceptions, or their cross-level alignment better explains early-career proactivity. Third, the online survey procedure and measurement model should be strengthened in future research. Although the present study screened responses and reported additional measurement diagnostics, future studies should pre-register exclusion rules, retain itemized quality-control flags, use independent supervisor-rated proactive behavior, and further refine the locus-of-control measure to separate internal-control beliefs from reverse-keyed external-control wording. Fourth, future studies should examine mediating mechanisms and additional boundary conditions. Intrinsic work values may influence proactive behavior through autonomous motivation, psychological meaningfulness, role-breadth self-efficacy, psychological empowerment, person-organization fit, career adaptability, or future work self salience. Testing these mechanisms would help explain not only when intrinsic work values matter, but also how they become proactive behavior in daily work.

## 6 Conclusion

This study developed and tested a value-based account of proactive behavior among Chinese early-career employees. The findings indicate that intrinsic work values were positively associated with proactive behavior, suggesting that employees who view work as a source of learning, challenge, achievement, and meaningful self-development are more likely to report self-starting and future-oriented work behavior. At the same time, this association was not uniform across contexts and individuals. Perceived employment relations climate attenuated the association, indicating that a favorable relational climate may make proactive behavior broadly legitimate and therefore reduce the extent to which proactivity depends on individual differences in intrinsic values. Internal locus of control strengthened the association, suggesting that growth-oriented values are more likely to be enacted when employees believe that their own actions can influence work outcomes. Overall, the study supports a person-context account in which early-career proactivity reflects the joint role of value-based motivation, situational cues, and perceived agency.

## Data Availability

The raw data supporting the conclusions of this article will be made available by the authors, without undue reservation.
